# Primary Ewing sarcoma/primitive neuroectodermal tumor of the renal pelvis: a case report

**DOI:** 10.1186/1477-7819-12-293

**Published:** 2014-09-22

**Authors:** Zhihong Liu, Xianding Wang, You Lu, Libo Chen, Yiping Lu

**Affiliations:** Department of Urology, West China Hospital, Sichuan University, Number 37, Guoxue Alley, Chengdu, Sichuan, 610041 The People’s Republic of China; Department of paediatrics, West China Second University Hospital, Chengdu, Sichuan Province 610041 China

**Keywords:** Ewing sarcoma, Diagnosis, Primitive neuroectodermal tumor, Renal pelvis, Therapeutics, Ureteroscopic biopsy, Nephroureterectomy

## Abstract

Ewing sarcoma/primitive neuroectodermal tumor (ES/PNET) is a childhood malignancy, typically occurring in the bone and rarely in any other part of the body. We herein present a case of ES/PNET of the renal pelvis. A 37-year-old male patient presented with a chief complaint of pain in the left flank and gross hematuria. The tumor had caused moderate hydronephrosis, and ureteroscopic biopsy findings were highly suspicious of sarcoma. Subsequently, radical nephroureterectomy was performed. On the basis of the pathological and cytogenetic findings, a final diagnosis of primary ES/PNET of left renal pelvis was made. Adjuvant chemotherapy with adriamycin and ifosfamide was initiated as ES/PNET often exhibits aggressive biological behavior. The patient was disease-free at his last regular follow-up visit 18 months after the surgery. To our knowledge, this is the first reported case of primary ES/PNET of the renal pelvis.

## Background

Ewing sarcoma/primitive neuroectodermal tumor (ES/PNET) is the second most common form of bone malignancy (after osteosarcoma) occurring in childhood [1]. However, ES/PNET of extra-skeletal origin is an extremely rare entity. With the exception of ES/PNET arising from the renal parenchyma, ES/PNET of the renal pelvis has never been reported in the literature. To help establish the biological nature of primary ES/PNET of the renal pelvis, we present the clinical, diagnostic, and therapeutic aspects of this rare neoplasm along with a review of the literature.

## Case presentation

**Figure 1:**
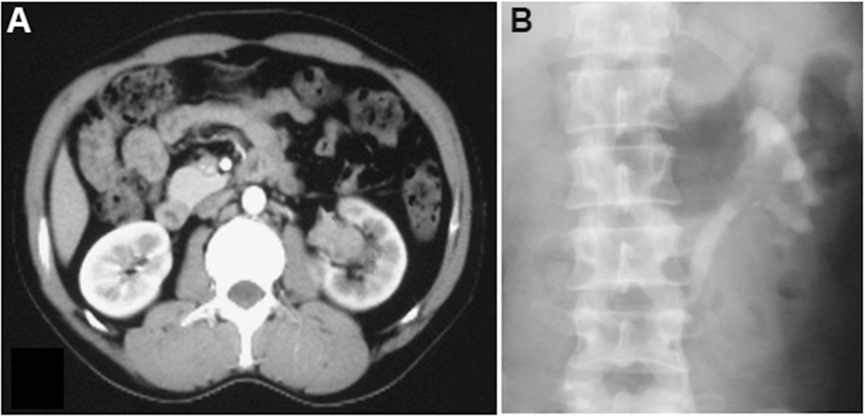
**Contrast-enhanced CT demonstrating a 4-cm soft-tissue mass occupying the dilated left renal pelvis. (A)** Enhanced CT. **(B)** Left retrograde pyelography.

A 37-year-old male patient was admitted with a chief complaint of intermittent pain in the left plank and gross hematuria. His past medical history and findings of a physical examination were unremarkable. Results of his blood chemistry and routine blood tests were within the normal range. Urine cytology showed no signs of malignancy. Ultrasonography demonstrated a 4-cm hypoechoic mass occupying the dilated left renal pelvis, the presence of which was subsequently confirmed by enhanced computed tomography (CT) (Figure 1) and left retrograde pyelography. Cytology of the washout fluid obtained from the retrograde catheterization was also negative for malignancy. Further clinical investigations showed no evidence of metastasis. A preoperative ureteroscopic biopsy performed, with findings highly suspicious of sarcoma. The patient subsequently underwent open, left radical nephroureterectomy with excision of the bladder cuff. No intraoperative or postoperative complications developed.

**Figure 2:**
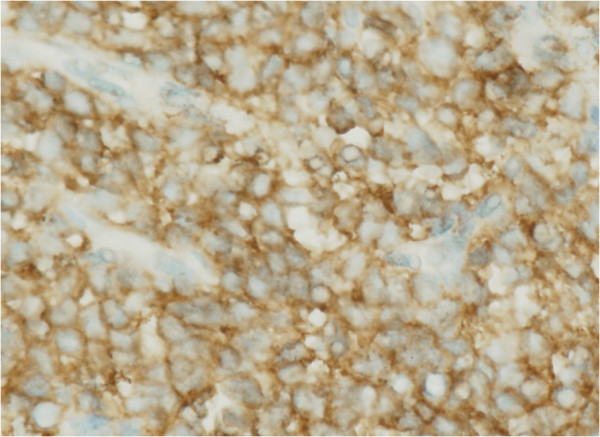
**Immunohistochemistry showed intense positive membranous CD99 staining.**

Macroscopic examination showed that a 4.0 × 2.3 × 1.5 cm soft tissue mass was situated in the renal pelvis, with lobulated contours, a gray-white cut surface, and an intact renal parenchyma. Microscopic examination revealed a small round cell tumor with focal necrosis, infiltrating the smooth muscle layer of the renal pelvis and without renal parenchyma involvement. On immunohistochemistry, the tumor cells stained positive for CD99 (Figure 2), CD56, S-100, and vimentin; focally positive for epithelial membrane antigen and Ki-67; and negative for Bcl-2, cytokeratin 7, cytokeratin 20, desmin, WT-1, muscle specific actin, and Syn. On the basis of these findings, a pathological diagnosis of primary ES/PNET of the left renal pelvis was made. The diagnosis was further confirmed by fluorescence in situ hybridization (FISH) to detect the presence of EWS/FLI-1 fusion products. Adjuvant chemotherapy with adriamycin and ifosfamide was administered as ES/PNET often exhibit highly aggressive biological behavior. The patient was in excellent physical and mental condition without recurrence or metastasis as confirmed by careful follow-up at 18 months postoperatively.

## Conclusion

During the past two decades, nine cases of primary sarcoma of the renal pelvis have been described in the English literature: six of leiomyosarcoma, two of rhabdomyosarcoma, and one of cystic embryonal sarcoma (Table 1) [2–10]. To our knowledge, this is the first case of primary ES/PNET of the renal pelvis to be reported in the literature. Although being a rare entity, sarcoma should be included in the preoperative differential diagnosis of renal pelvis tumors. However, preoperative diagnosis of sarcoma is often difficult because of its rare incidence, non-specific clinical presentation, and radiological features (similar to those of urothelial carcinoma). Tsai et al. [8] and Chow et al. [9] reported the use of fine-needle aspiration biopsy with good results for primary renal pelvis sarcoma. Furthermore, for tumors of the upper urinary tract, ureteroscopic biopsy may be useful in establishing a more accurate preoperative diagnosis and deciding on the surgical modality, as was performed in the case reported here.

**Table 1:** **Sarcoma of the renal pelvis reported in the English literature during the past two decades** [2–10]

First author/Year	Age (yr)/Sex	Symptoms & signs	Side	Size (cm)	Therapy	Histological subtype	Follow-up
Chow 1994	56/F	Abdominal distension	R	15	RN, thromb-ectomy	Leiomyosarcoma	2 yr/DOM
Ito 2000	12/M	Abdominal pain, massive bleeding	R	NA	SN	Cystic embryonal sarcoma	26 yr/NED
Moudouni 2001	41/M	Flank pain, gross hematuria	L	2	RNU	Leiomyosarcoma	8 yr/NED
Kren 2003	49/F	Flank pain	R	1.8	RNU	Rhabdomyo-sarcoma	2 yr/NED
Minami 2004	54/M	Bowel discomfort	L	8	RN	Leiomyosarcoma	6 mo/NED
Kartsanis 2006	44/M	Gross hematuria	L	5	RNU, PL	Leiomyosarcoma	3 yr/NED
Tsai 2006	77/F	Gross hematuria, voiding difficulty, flank tenderness	R	2	RN	Rhabdomyo-sarcoma	5 mo/Alive
Chung 2007	42/F	Asymptomatic	L	5	RN	Leiomyosarcoma	NA
Dhamne 2009	60/M	Urinary frequency, weakness, weight loss	R	10	RN	Leiomyosarcoma	6 mo/NED
Our case 2012	37/M	Flank pain, gross hematuria	L	4	RNU, chemo-therapy	Ewing’s sarcoma	4 yr/NED

Histologically, lymphoblastic lymphoma, neuroblastoma, rhabdomyosarcoma, poorly differentiated synovial sarcoma, and Wilms tumor are included in the differential diagnosis of ES/PNET because they are all small round cell tumors. High levels of universal membranous CD99 expression are seen in ES/PNET cells, but this is not specific for ES/PNET. Thus, a broad immunohistochemistry panel may aid in differentiating these unique entities, even though this is sometimes difficult. Recently, molecular techniques have been increasingly used to confirm the diagnosis of ES/PNET, as it is one of the few solid tumors for which underlying chromosomal translocations have been described [11]. The most common translocation is the t(11, 22) (q24; q12), detected in more than 85% of ES/PNET by FISH or reverse transcription-polymerase chain reaction. Accurate diagnosis is essential, as these tumors require different treatment strategies.

As ES/PNET rarely spreads to lymph nodes, the single most important factor considered when planning initial treatment is the extent of disease at presentation, with the lungs being the most common site of metastasis. Initial presentation is the main factor that influences prognosis. The 5-year survival rate associated with extraskeletal ES/PNET in adults is 60% and 33% for non-metastatic and metastatic disease [1, 12], respectively. Surgical resection may be the treatment of choice for local control in ES/PNET. However, ES/PNET often results in rapid recurrence or metastasis, even after complete resection. Therefore, patients with ES/PNET may require multidisciplinary management, combining systemic neoadjuvant and/or adjuvant chemotherapy with local control measures (surgery and/or radiation). Ifosfamide-based protocols appear to confer a survival advantage in ES/PNET patients [12, 13]. Novel molecular-targeted therapies for the treatment of ES/PNET have now transitioned from the laboratory to the clinical setting. In a recent phase I trial of a fully human IgG2 monoclonal antibody targeting the insulin-like growth-factor-1 receptor, figitumumab, a number of patients with refractory, advanced ES/PNET responded unexpectedly well [14]. Such patients should be informed about relevant clinical trials and encouraged to enter these trials. Due to the rarity of this condition, the impact of different treatment modalities (simple nephrectomy *vs.* radical nephrectomy *vs.* radical nephroureterectomy with or without chemotherapy and/or radiotherapy) on outcomes cannot be evaluated until more cases have been reported.
